# Extraosseous Ewing Sarcoma in a 28-Year-Old Male: A Case Report and Literature Review

**DOI:** 10.7759/cureus.63910

**Published:** 2024-07-05

**Authors:** Vladimir Osadchyi, James Vredenburgh

**Affiliations:** 1 Internal Medicine, University of Connecticut, Farmington, USA; 2 Hematology and Oncology, Saint Francis Hospital, Hartford, USA

**Keywords:** extraosseous, extraosseous ewing sarcoma, extraskeletal ewing sarcoma, oncology case reports, molecular oncology, hematology-oncology, ewing sarcoma family of tumors (esft), rare cancer, ewing sarcoma (es)

## Abstract

Ewing sarcoma (ES) is an uncommon and highly aggressive bone malignancy that predominantly occurs in children and young adults. Extraosseous Ewing sarcoma (EES), an even rarer variant, can present in the soft tissues instead of bone. In this case report, we detail a previously healthy 28-year-old male presenting with an isolated enlarged left inguinal lymph node, subsequently diagnosed as EES. The patient presented with a three-month history of a non-tender, gradually enlarging lump in the left groin. Fine needle aspiration revealed a small round blue cell tumor with a high Ki-67 score, and subsequent excisional biopsy identified a rare genetic fusion mutation. Postoperative positron emission tomography (PET)/computed tomography (CT) scan did not show any fludeoxyglucose F18 (FDG) uptake lesions to suggest residual malignancy. The patient is currently awaiting chemotherapy. Throughout the discussion of this case, we highlight the importance of considering EES in the differential diagnosis of isolated lymph node enlargement, the role of genetic testing in diagnosis, and the treatment modalities offered.

## Introduction

Ewing sarcoma (ES) is a rare and aggressive type of bone cancer that primarily affects children and young adults. It belongs to a group of tumors known as the Ewing sarcoma family of tumors (ESFT). ES typically arises in the long bones such as the femur, tibia, and humerus, but can also be found in the pelvis, ribs, and spine. When found in bones, ES typically arises from the diaphysis, and very rarely does it involve the epiphysis. Occasionally, ES can develop in soft tissues, including lymph nodes, which is classified as extraosseous Ewing sarcoma (EES).

A hallmark of ES is the presence of a specific chromosomal translocation, most commonly t(11;22)(q24;q12), involving the EWSR1 (Ewing Sarcoma Breakpoint Region 1) gene on chromosome 22 and the FLI1 (Friend Leukemia Virus Integration 1) gene on chromosome 11 [1]. This translocation results in the fusion of the EWSR1 and FLI1 genes, producing an abnormal protein that contributes to cancer development. The EWSR1-FLI1 fusion accounts for approximately 90% of ES cases and predominantly presents with skeletal tumors [1].

Various other genetic mutations characterize the remaining 10% of ES cases. In this case report, we detail a previously healthy 28-year-old male who presented with an isolated enlarged left inguinal lymph node and was later diagnosed with EES, which featured a rare genetic fusion mutation.

## Case presentation

A 28-year-old previously healthy male presented with a three-month history of a progressively enlarging lump in his left groin region. The lump was non-tender, without overlaying skin changes, and was not warm to the touch. The mass measured approximately 2.8 cm in size and was soft and rubbery. The patient did not report any other lumps, fevers, chills, night sweats, fatigue, or changes in appetite or weight.

**Figure 1:**
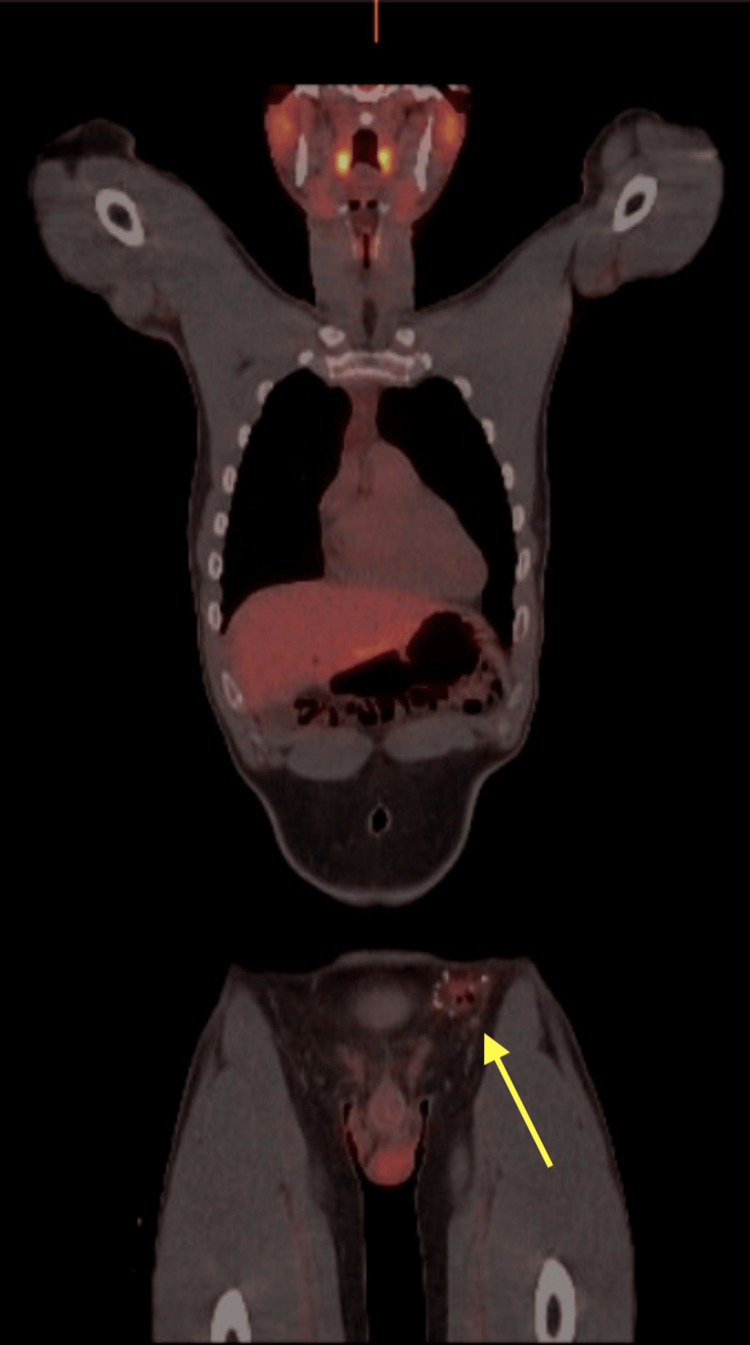
PET of the skull base to mid-thigh There is mild increased FDG uptake within the anterior left groin region consistent with postoperative inflammatory changes which is indicated by the yellow arrow in the figure. No additional abnormal FDG uptake in the neck, chest, abdomen, or pelvis was seen, and there were no osseous lesions identified PET: positron emission tomography; FDG: fludeoxyglucose F18

**Figure 2:**
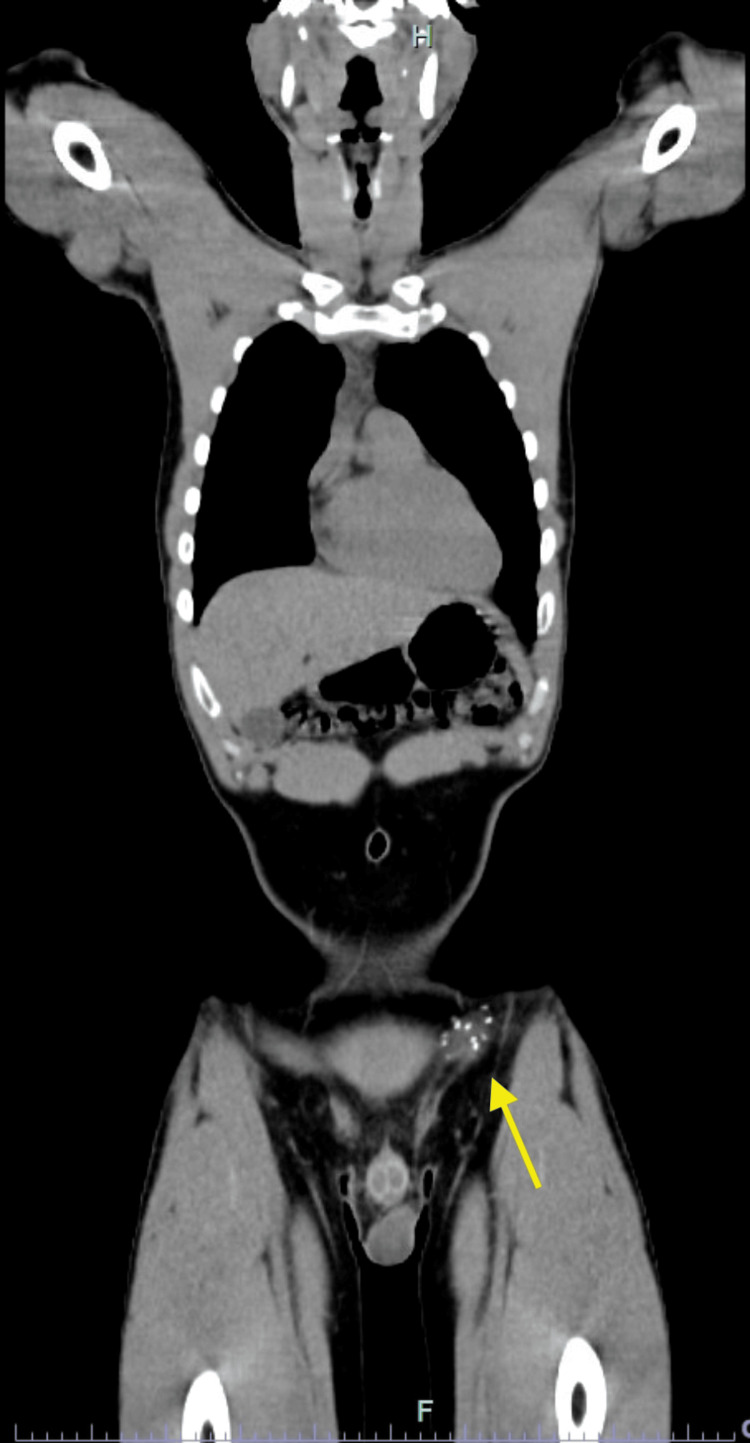
Noncontrast CT imaging of the skull base to mid-thigh Noncontrast CT imaging demonstrates clear lungs, no adenopathy, vascular phleboliths in the pelvis, and surgical clips in the left groin which are illustrated by the yellow arrow in the figure CT: computed tomography

Initial fine needle aspiration (FNA) of the mass revealed a small round blue cell tumor with a high Ki-67 score. An excisional biopsy of the 2.8 cm mass was performed, and next-generation sequencing identified a t(22;2)(22q12.1;2q35) fusion consistent with an EWSR1-FEV mutation, a rare alteration associated with EES with aggressive behavior. A nuclear medicine positron emission tomography (PET)/computed tomography (CT) scan from the skull base to mid-thigh showed no evidence of fludeoxyglucose F18 (FDG) uptake lesions to suggest the presence of malignancy and only indicated postoperative changes in the left groin (Figure 1 and Figure 2).

The patient recovered well post-excisional biopsy and is currently undergoing sperm banking and echocardiography testing in preparation for combined chemotherapy and radiotherapy. 

## Discussion

There is no specific clinical manifestation of EES. The most prevalent symptom includes a rapidly growing mass that may be accompanied by localized pain [2]. The imaging characteristics of EES are non-specific and largely depend on where the mass is located and the type of imaging. On X-ray, EES may present as a soft tissue mass with varying degrees of calcification. On CT, EES typically appears as a well-defined soft tissue mass with heterogeneous density. The tumor may also have areas of necrosis, hemorrhage, or calcification [3,4]. On magnetic resonance imaging (MRI), EES typically appears as a hyperintense mass on T2-weighted images and may have varying signal intensity on T1-weighted images. The tumor often shows enhancement with contrast administration [5].

A definitive diagnosis of EES is made via biopsy and pathological examination of the specimen. Typical findings associated with EES are small, round, blue cells which are a hallmark histological feature of ES, both skeletal and extraosseous. Nuclear features include finely dispersed chromatin and prominent nucleoli. Occasionally, Homer-Wright rosettes may be observed, which are circular arrangements of tumor cells around a central space. Additional testing, such as immunohistochemistry, may be carried out on biopsy samples. ES cells typically express markers such as CD99 and FLI1. Finally, molecular testing, such as fluorescence in situ hybridization (FISH) or reverse transcription-polymerase chain reaction (RT-PCR), is frequently employed to detect the characteristic translocations [6].

The literature comparing patient demographics between skeletal ES and EES found the following: patients with EES tend to have a higher mean age, showing a bimodal distribution. EES is more frequently observed in individuals over 35 years old and under five years old compared to those with skeletal tumors. Patients with EES are also less likely to be male or White. Finally, the tumors observed in EES are more commonly seen in axial locations but less likely to arise from the pelvis specifically [7].

The treatment of skeletal ES and EES are largely the same; however, some literature suggests that surgery may play a more important role in EES compared to skeletal ES, with complete resection increasing the likelihood of survival [8,9].

The treatment for ES, both skeletal and extraosseous, typically involves a multidisciplinary approach, combining surgery, chemotherapy, and sometimes radiation therapy. The specific treatment plan is individualized based on factors such as the location and size of the tumor, the extent of its spread, and the overall health of the patient. In North America, the typical treatment of ES includes chemotherapy treatment of alternating vincristine-doxorubicin-cyclophosphamide and ifosfamide-etoposide cycles with management of the primary tumor site with surgery and/or radiation [10].

## Conclusions

This case report illustrates a rare presentation of EES in a 28-year-old male, who presented with an isolated enlarged left inguinal lymph node. The diagnosis was confirmed through FNA and subsequent excisional biopsy, revealing a rare t(22;2)(22q12.1;2q35) fusion. This case emphasizes the importance of considering EES in the differential diagnosis of isolated lymph node enlargements, even in the absence of specific clinical manifestations or systemic symptoms. Early recognition and appropriate genetic testing are critical for accurate diagnosis and timely initiation of treatment, which is vital for improving patient outcomes. The findings also underscore the potential aggressive nature of EES and the need for a multidisciplinary treatment approach, including surgery, chemotherapy, and potentially radiation therapy. Further research is necessary to better understand the genetic landscape of EES and to refine treatment protocols for optimizing patient survival and quality of life.

## References

[1] Tsuda Y, Dickson BC, Swanson D (2020). Ewing sarcoma with FEV gene rearrangements is a rare subset with predilection for extraskeletal locations and aggressive behavior. Genes Chromosomes Cancer.

[2] Maheshwari V, Siddiqui F, Adreena Adreena, Sherwani R, Jain A, Alam K (2008). Extraskeletal Ewings sarcoma- a case report. Internet Journal of Orthopedic Surgery.

[3] Vanel D, Contesso G, Couanet D, Piekarski JD, Sarrazin D, Masselot J (1982). Computed tomography in the evaluation of 41 cases of Ewing's sarcoma. Skeletal Radiol.

[4] O'Keeffe F, Lorigan JG, Wallace S (1990). Radiological features of extraskeletal Ewing sarcoma. Br J Radiol.

[5] Frouge C, Vanel D, Coffre C, Couanet D, Contesso G, Sarrazin D (1988). The role of magnetic resonance imaging in the evaluation of Ewing sarcoma. A report of 27 cases. Skeletal Radiol.

[6] Galyfos G, Karantzikos GA, Kavouras N, Sianou A, Palogos K, Filis K (2016). Extraosseous Ewing sarcoma: diagnosis, prognosis and optimal management. Indian J Surg.

[7] Applebaum MA, Worch J, Matthay KK, Goldsby R, Neuhaus J, West DC, Dubois SG (2011). Clinical features and outcomes in patients with extraskeletal Ewing sarcoma. Cancer.

[8] Rud NP, Reiman HM, Pritchard DJ, Frassica FJ, Smithson WA (1989). Extraosseous Ewing's sarcoma. A study of 42 cases. Cancer.

[9] Covelli HD, Beekman JF, Kingry RL (1980). Extraskeletal Ewing's sarcoma: prolonged survival with recurrence after operation. South Med J.

[10] Womer RB, West DC, Krailo MD (2012). Randomized controlled trial of interval-compressed chemotherapy for the treatment of localized Ewing sarcoma: a report from the Children's Oncology Group. J Clin Oncol.

